# Can auditory warning signals normalize eye movements in children with ADHD?

**DOI:** 10.1007/s00787-020-01484-w

**Published:** 2020-02-01

**Authors:** Johan Lundin Kleberg, Matilda A. Frick, Karin C. Brocki

**Affiliations:** 1 Department of Clinical Neuroscience, Centre for Psychiatry Research, Karolinska Institutet and Stockholm Health Care Services, Region Stockholm, Gävlegatan 22, 113 30 Stockholm, Sweden; 2 Uppsala Child and Baby Lab, Department of Psychology, Uppsala University, Box 1225, 751 42 Uppsala, Sweden

**Keywords:** ADHD, Pupil dilation, Gap/overlap task, Saccades, Eye tracking

## Abstract

Attenuated baseline arousal has been hypothesized to underlie symptoms of attention deficit/hyperactivity disorder (ADHD). A behavioral signature of reduced baseline arousal is an increased beneficiary effect of warning signals in reaction tasks. This paradoxical effect is believed to be caused by a temporary increase in arousal induced by warning signals. In a preregistered study, we tested the hypothesis that children with high levels of ADHD symptoms would be hyperresponsive to warning signals in a well-established visual attention task (the gap/overlap paradigm). Previous studies using this task have found slower and more variable saccadic reaction times in children with ADHD compared to typically developing children, suggesting that these eye movement metrics are candidate biomarkers. We examined 71 children, of which 1/3 had a diagnosis of ADHD, using both dimensional analyses and group comparisons. Previously reported findings of reduced saccadic latency and increased latency variability were replicated. Importantly, saccadic latency was normalized by auditory warning signals. Analyses of pupil dilation, a physiological index of arousal and locus coeruleus-noradrenergic activity, confirmed that warning signals led to enhanced arousal. Our findings are novel and contribute to our understanding of arousal and attention in ADHD and have implications for treatment and interventions.

**Electronic supplementary material:**

The online version of this article (10.1007/s00787-020-01484-w) contains supplementary material, which is available to authorized users.

## Introduction

Attention deficit/hyperactivity disorder (ADHD) is one of the most prevalent childhood onset mental disorders, affecting approximately 5% of children worldwide [1]. Contemporary models define ADHD as consisting of two highly correlated but dissociable symptom dimensions: hyperactivity/impulsivity and inattention [2]. Symptoms of ADHD are continuous, with the full syndrome constituting the extreme end of phenotypic traits and genetic vulnerability factors found in the general population [3, 4]. ADHD is associated with impairments in numerous cognitive functions, including working memory, sustained attention, inhibition, and other executive functions, but the neuropsychological profile of ADHD is heterogeneous [5–7] and a significant proportion of children with ADHD are not impaired on standard neuropsychological tests.

Studies of eye movements can contribute to a better understanding of the underlying mechanisms of the disrupted arousal processes associated with ADHD. There are at least two reasons to pursue this line of research. Firstly, eye movements are closely linked to attention, since information reaching the fovea is highly prioritized for further cortical processing. Secondly, the mechanisms underlying saccade and fixation metrics are relatively well characterized, meaning that atypical performance can be informative about implicated brain functions [8, 9]. Previous studies have linked ADHD to slower and more variable saccadic reaction times [10, 11], and difficulties inhibiting reflexive gaze shifts [10, 12, 13]. Impaired saccadic programming has also been found in ADHD [14]. A commonly studied measure of saccadic programming efficiency is the *gain*, defined as the difference between the observed distance (amplitude) of a saccade and the optimal distance to its intended target. These oculomotor abnormalities may be partly normalized by pharmacological treatment [15], meaning that eye movements are promising as biomarkers.

### Arousal and ADHD

Multiple processes determine how eye movements are planned and executed, with one mechanism being level of arousal (e.g. [16]). An influential theory links ADHD to impaired baseline (tonic) arousal [17–19]. Reduced tonic arousal could be a direct cause of inattention symptoms, including variable performance and failure to react to novel stimuli [20–22]. Indirectly, hypo-arousal has also been suggested to lead to hyperactivity, which could be an autoregulatory attempt to normalize tonic arousal by increasing sensory stimulation [23, 24]. The hypo-arousal theory of ADHD is supported by studies using both central nervous system and peripheral indices of arousal at rest and during various cognitive tasks [25–27]. Psychostimulants, the preferred pharmacological treatment for ADHD, may have their therapeutic results by affecting the arousal related neurotransmitter systems dopamine and noradrenaline [28]. At a behavioral level, arousal can be increased by long or short periods of sensory stimulation, such as extended periods of white noise or time-locked warning signals. These manipulations are particularly effective in influencing behavior and performance in populations with low levels of baseline (tonic) arousal. For example, the effects of warning signals are enhanced after sleep deprivation [16] or pharmacological manipulations, which decrease arousal [29]. Changes in cognition or behavior caused by a short-term increase in arousal after warning signals are termed *phasic alerting* effects, and mediated by activity in the brains locus coeruleus-noradrenergic (LC-NE) system [17, 30, 31].

Given that the hypo-arousal theory of ADHD is correct, phasic alerting effects on eye movements should be enhanced in individuals with either an ADHD diagnosis or high levels of ADHD symptoms. Importantly, this prediction has not been tested before. Studies using other methodologies have suggested atypical responsiveness to a range of sensory stimuli in individuals with ADHD. For example, studies using event-related potentials (ERPs) have reported atypical neural responsiveness to warning signals in cognitive tasks [32, 33]. There is also evidence that reaction time variability can be reduced in individuals with high levels of ADHD symptoms by increased pace of stimulus presentation or external rewards, manipulations which may have their effect through an increase in arousal [34, 35]. Cognitive performance can be improved by exposure to prolonged periods of white noise in children with ADHD [36], but it is not clear whether this improvement is driven by an increase in arousal [37].

### The gap/overlap paradigm

**Fig. 1:**
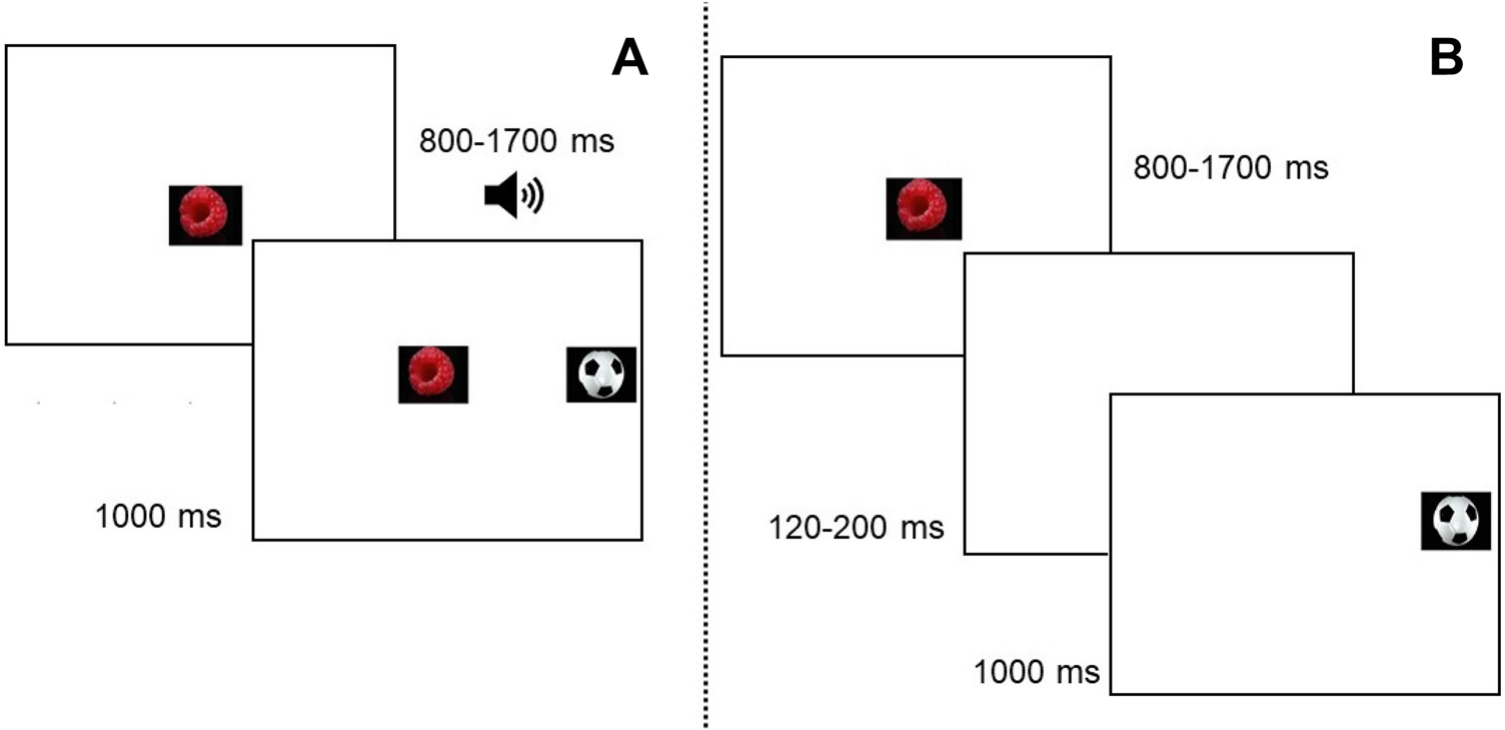
Example of stimuli in the overlap condition (**a**), and the gap condition (**b**). The central stimulus was presented during a variable interval ranging between 800–1700 ms. In the gap condition (**b**), the central stimulus was extinguished before the onset of the peripheral stimulus, creating a temporal gap ranging between 120–200 ms. Auditory alerting cues were presented at variable time intervals ranging between 500 and 0 ms before the onset of the peripheral stimulus on 50% of the overlap trials

The present study tested the effects of arousal on saccadic latencies in relation to ADHD symptoms using the gap/overlap paradigm. This paradigm is well established and has been used extensively in research on ADHD and other neurodevelopmental disorders [38–40]. In the basic version of the task (see Fig. 1), participants initially fixate a stimulus in the center of a screen, and then initiate a gaze shift to an upcoming peripheral stimulus. Saccadic reaction times are measured under two conditions: gap trials, where the central target is extinguished before the onset of the target (creating a temporal “gap”) and overlap trials, where the central stimulus remains on the screen during the onset of the target (creating a temporal “overlap”). Gap trials typically lead to shorter saccadic reaction times than overlap trials, a phenomenon called the *gap effect*. Previous studies have shown that the gap effect results from a combination of two phenomena [39, 41]. First, the focus of visual attention has to be disengaged from the central stimulus during overlap trials, which is associated with a time cost. Secondly, the disappearance of the central stimulus during gap trials functions as a spatially non-specific warning cue which increases wakefulness and arousal and decreases reaction time. The gap effect is considerably decreased, but not eliminated, if an alerting cue is presented shortly before the onset of the peripheral stimulus in the overlap condition [42–44]. The warning signal component of the gap effect can therefore be examined by comparisons between cued and non-cues overlap trials. As noted previously, the effects of alerting cues are closely linked to LC-NE functioning. An indirect physiological marker of LC-NE activity is the pupil dilation response [45, 46]. In the present study, pupil dilation was used as an index of arousal induced by phasic alerting cues. The ADHD phenotype is highly overlapping with symptoms of externalizing disorders, and particularly oppositional defiant disorder (ODD) [47]. Given this overlap, it is important to examine whether potential markers for ADHD symptoms are disorder specific, or better explained by coexisting symptoms of externalizing disorders. Therefore, symptoms of ODD were added as covariates in the analyses.

### Aims and hypotheses

In light of previous studies, we expected ADHD symptoms to be linked to longer saccadic latencies and less optimal gain in the absence of phasic alerting cues. The main hypothesis of the present study was that ADHD symptoms would be associated with an enhanced effect of phasic alerting cues. In statistical terms, this would result in (1) an association between ADHD symptoms and longer saccadic latencies and less optimal gain in the absence of alerting cues, (2) an interaction effect between ADHD symptoms and condition (silent or cued), and (3) a weaker association between ADHD symptoms and saccade metrics after phasic alerting cues than on silent trials. To examine the mechanisms underlying the hypothesized phasic alerting effect, we compared the pupil dilation response between cued and silent overlap trials. If the phasic alerting effect stems from an increase in arousal, alerting cues would be expected to elicit a pupil dilation response.

In light of previous studies showing that ADHD is linked to increased intra-individual response variability, the variability of the saccadic latency and gain measures were also examined. Both dimensional analyses and group comparisons between children with ADHD and typical development were conducted. Since ODD and ADHD are highly comorbid conditions, ODD symptom level was added as covariate in all dimensional analyses, but we did not hypothesize a relation between ODD symptoms and phasic alerting effects.

### Pre-registration

The analysis plan was pre-registered in the open science framework [registration number removed for anonymization].

### Ethical approval

The study was approved by the regional ethics committee of Uppsala, Sweden, and the study was performed in accordance with the ethical standards laid down in the 1964 Declaration of Helsinki and its later amendments.

## Methods

### Participants

The final sample consisted of 71 children (27% female) of which 24 had received a diagnosis of ADHD.

### Children with ADHD

Families of children with ADHD were contacted through outpatient clinics and advertising in newspapers and social media. Initially, 34 children with ADHD and their families initially agreed to participate. Of these, 10 were excluded for the following reasons: no questionnaires were handed in (*n* = 1), the child was lacking valid data from all conditions (*n* = 1, see “Recording and processing of eye-tracking data”), the child had taken psychostimulant medication at the day of testing (*n* = 8). Ten included children with ADHD were treated with stimulant or nonstimulant medication for ADHD, but medication was withdrawn at the day of testing. Parents confirmed that the child had received a diagnosis of ADHD from a clinical psychologist or psychiatrist in regular care and specified the clinic and year of diagnosis. Of the included children, one had diagnosis of ADHD-PI, one had ADHD-NOS, and 22 had the combined presentation. Parent ratings confirmed symptom levels within the range of clinical concern according to the SNAP-IV [48] in all but two cases. These children were included in the analyses, but we conducted exploratory analyses after excluding them. This did not change any of the results. Comorbid diagnoses according to medical records or parental report were dyslexia (*n* = 2), speech and language disorder (*n* = 1), and developmental coordination disorder (*n* = 1). None of the children had received a formal diagnosis of ODD, but teacher and parent ratings on the SNAP-IV indicated that seven children with ADHD (29%) fulfilled criteria for ODD, defined as the mean of parent and teacher ratings of 2 or higher (indicating that the specified behavior is “pretty much” or “very much” characteristic for the child) on four or more ODD symptoms. These numbers are consistent with published prevalence estimates [47].

In addition to the children with an ADHD diagnosis, a group of typically developing children was recruited. One thousand families in the local area with children in the 8–12 years age range were randomly selected from the population registry and contacted by mail. One hundred and sixteen interested parents responded to an on-line survey, and children who matched the diagnosed children on age, sex, and when possible socio-economic status (SES) were invited and took part in the study. In total, 48 children were invited and tested. Of these, one child had no valid data due to equipment failure. None of the typically developing children had a psychiatric disorder according to parental report or questionnaires, but teacher ratings on the Strength and Difficulties Questionnaire (SDQ) [49] and SNAP-IV [50] were in the clinical range for three participants (ADHD-C, conduct, and emotional problems, *n* = 1; ADHD-PI, *n* = 1; emotional problems, *n* = 1), defined as scores above the 90th percentile of the national norms [51]. Given the aim to study symptom dimensions across the whole range, these participants were included in the analyses.

Parental education was graded on a scale from 1 (representing 9 years of schooling or less) to 6 (representing a master or doctoral degree). Income was also graded on a six-point Likert scale, with 1 corresponding to an annual income less than approximately 10,500 USD in the local currency, and 6 to approximately 52,500 USD or more). SES was operationalized as the mean of both parents’ education and income levels. IQ was estimated as the mean scaled score of the two sub tests Block design and Information from the Wechsler Intelligence Scale for Children (WISC, Fourth Edition) [52]. As can be seen in Table 1, no significant group differences between children with and without ADHD were found for SES, IQ, age, or gender proportion.

**Table 1:** Demographics, clinical information, and number of valid trials

	Full sample		ADHD		Typically developing		*p*
	M (SD)	Range	M (SD)	Range	M (SD)	Range	
Sex (% female)	0.27 (0.45)	0.00–1.00	0.33 (0.48)	0.00–1.00	0.23 (0.43)	0.00–1.00	0.379
Age	10.48 (1.39)	8.00–13.00	10.55 (1.65)	8.00–13.00	10.44 (1.26)	8.25–12.17	0.761
SES	4.18 (0.97)	2.00–5.50	3.88 (1.00)	2.00–5.50	4.34 (0.93)	2.25–5.50	0.060
IQ	9.81 (2.42)	3.50–15.50	9.81 (3.30)	3.50–15.50	9.81 (2.00)	5.50–14.00	0.993
*Symptoms*							
ADHD	0.74 (0.64)	0.00–2.44	1.48 (0.51)	0.67–2.44	0.37 (0.25)	0.00–1.31	< 0.001
ADHD-IN	0.83 (0.65)	0.00–2.56	1.54 (0.52)	0.56–2.56	0.46 (0.31)	0.00–1.22	< 0.001
ADHD-H/I	0.63 (0.63)	0.00–2.39	1.32 (0.58)	0.00–2.39	0.27 (0.26)	0.00–1.39	< 0.001
ODD	0.50 (0.52)	0.00–2.19	0.89 (0.64)	0.13–2.19	0.30 (0.28)	0.00–1.13	< 0.001
SDQ emotional Problems	1.20 (1.89)	0.00–7.00	1.88 (2.03)	0.00–6.00	0.89 (1.76)	0.00–7.00	0.073
SDQ peer problems	1.28 (1.81)	0.00–7.00	2.71 (2.23)	0.00–7.00	0.62 (1.09)	0.00–4.00	< 0.001
SDQ hyperactivity	4.19 (2.36)	2.00–10.00	5.88 (2.47)	2.00–9.00	3.41 (1.88)	2.00–10.00	< 0.001
SDQ prosocial	12.13 (2.43)	6.00–15.00	10.47 (2.40)	6.00–15.00	12.89 (2.07)	8.00–15.00	< 0.001
SDQ total problems	7.83 (6.30)	2.00–25.00	13.06 (6.82)	2.00–24.00	5.43 (4.34)	2.00–25.00	< 0.001
*Valid trials*							
Gap	13.99 (3.28)	5.00–19.00	12.61 (3.31)	8.00–18.00	14.66 (3.07)	5.00–19.00	0.013
Overlap (silent)	13.06 (3.56)	5.00–18.00	11.36 (3.53)	5.00–18.00	13.87 (3.31)	6.00–18.00	0.006
Overlap (cued)	12.03 (3.37)	5.00–17.00	10.95 (2.40)	7.00–15.00	12.51 (3.65)	5.00–17.00	0.078

### Questionnaires

ADHD and ODD symptoms were measured with parental and teacher ratings on the SNAP-IV [50], which asks the informant to rate the degree of severity on each of the 18 ADHD symptoms and eight ODD symptoms listed in the DSM-V criteria on a four-point Likert scale. The SNAP-IV has good psychometric properties, with Cronbach’s α ranging from good to excellent (0.79–0.96) for different subscales [48].

Teachers completed the SDQ [49], a screening measure for emotional symptoms, conduct and peer problems and symptoms of hyperactivity/impulsivity. The SDQ also gives a total difficulties score, which is a composite measure for psychopathology. SDQ scores were not used in the main analysis, but as a screening measure for undetected psychopathology (see “Participants”). Teacher ratings for all of the scales were missing for 16 children (7 with ADHD, 22.5% of the final sample).

#### Experimental paradigm

The experimental paradigm was a modified version of the gap/overlap task (see Fig. 1). The paradigm included 60 trials, of which 20 were gap trials. Twenty trials were silent overlap trials, and 20 were overlap trials with an auditory alerting cue. Auditory cues had a variable onset and offset within the − 500 to 0 ms before the onset of the visual stimuli and a sound pressure level ranging between 65–75 db SPL. Fifty percent of the auditory cues were naturalistic sounds (spoken vowels), and 50% were simple beeps. The participants were instructed to attend to the screen, but were given no further instructions.

#### Recording and processing of eye-tracking data

Eye-tracking data were recorded with a corneal-reflection eye tracker (Tobii TX120, Tobii Inc, Danderyd, Sweden) at a sample rate of 60 HZ. Fixations were identified with an I-VT filter with velocity threshold set to 30°/s and window length set to 20 ms. *Saccadic latency* was defined as the latency in milliseconds to initiate a gaze shift from the central area of interest (AOI; see Fig. 1) to the peripheral target. *Saccadic gain* was defined as the absolute ratio of the observed Euclidean distance between the last point of fixation within the central AOI and the first fixation within the target AOI, and the optimal Euclidean distance between the last central fixation and the center of the target AOI (e.g. [53]). Thus, higher values reflect saccadic amplitudes more distant from the center of the AOI.

**Fig. 2:**
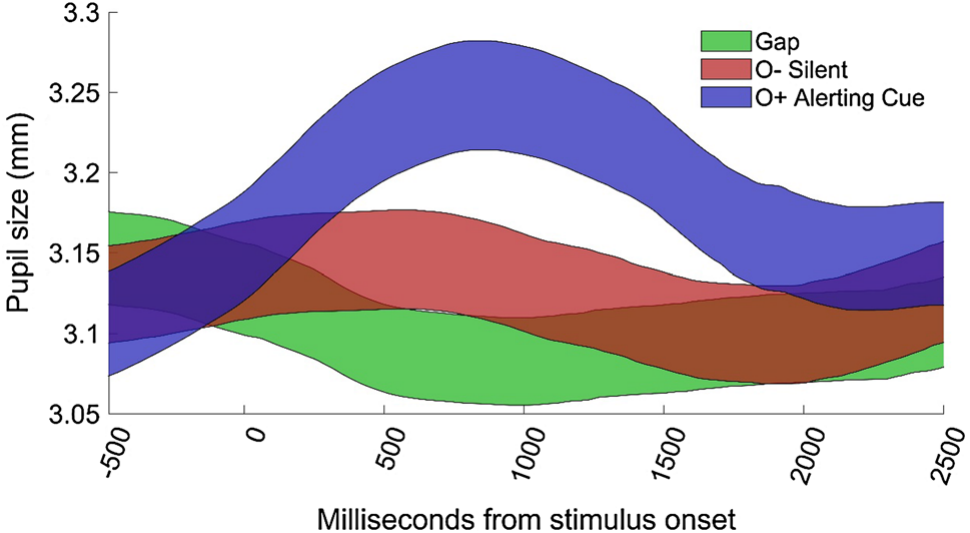
Average pupil dilation as a function of time in the gap for the total sample (*N* = 67) in the silent overlap (O-Silent) and, cued overlap (O+ alerting cue, conditions. Curves cover means ± 95% confidence intervals. Auditory cues had a variable onset and offset within the − 500 to 0 ms before the onset of the visual stimuli

Saccades were discarded if the point of gaze did not reach the peripheral target within 200 ms, or if the point of gaze was not within the central AOI during at least 25% of the 200 ms directly preceding the onset of the peripheral stimulus. Saccades with latencies < 120 ms were considered anticipatory, and were therefore discarded. Visual inspection of all recorded saccadic latencies indicated that the data were positively skewed due to a small number of very slow saccades. We removed saccades with latencies > 1000 ms (corresponding to the 97.5th percentile) from further analysis. The pupil signal was filtered using a moving median filter with a window size corresponding to 80 ms. A linear interpolation was applied over gaps in the pupil data shorter than 100 ms. Pupil-dilation amplitude was defined as the median size of the pupil during a 0–1500 ms time window after the onset of the visual stimuli, normalized to the median pupil size during a 333 ms (20 samples) baseline period. On silent trials, the baseline period was defined as the 1000–666 ms interval before stimulus onset (see Fig. 2). The baseline period after alerting cues was defined as the 333 ms directly before the onset of the auditory cues. Since pupil dilation amplitudes > 25% are physiologically unlikely [54], higher values were removed from analysis. Participants contributing less than five valid trials in any given condition were removed from this condition [gap: *n* = 4; overlap (cued): *n* = 5; overlap (silent): *n* = 4]. To test the validity of this approach, the analysis was conducted with the limit of trials set to a range of numbers to 3, 7, and 10 trials. This did not change the significance of any of the results.

#### Statistical analysis

The mean value of parent and teacher ratings of ADHD and ODD symptoms were analyzed when both sources were available (*n* = 55). For the other cases, parental ratings were used. Median values of saccadic latencies, saccadic gain, and pupil dilation were calculated for each participant and condition. Data were analyzed using generalized linear mixed effects models (GLMMs) with random intercepts for participant (equal to treating trials from the same individual as repeated measures), and random slopes for condition. The latency data were positively skewed, and the models were therefore fitted with a log link. As a measure of intraindividual variability, the interquartile range of saccadic gain and latency was calculated for each participant. The significance of main and interaction effects was tested with Analyses of variance (ANOVAs) with Satterthwaite approximated degrees of freedom. Bonferroni corrected *p *values are reported for all follow-up tests. The alpha level was set to 0.05. All analyses were conducted in MATLAB (Mathworks, Inc).Continuous variables were *z*-transformed for ease of interpretation. Sex and age were added as covariates in all analyses. ODD-symptom level was added as a covariate in the dimensional analyses. No significant main or interaction effects in the analyses involving ODD symptoms were found, and these are presented as *Supplementary analysis.* All significant results remained unchanged when ODD symptoms were excluded as covariate (see “Electronic supplementary material”).

Age was negatively related to saccadic latency in the silent overlap condition (*p* < 0.01). No other covariates were significantly linked to the dependent variables (all *p* > 0.10).

### Analysis plan

The analysis was conducted in two steps. First, we examined the gap effect by comparing overlap and gap trials. In a second step, we tested the preregistered hypotheses by comparing silent and cued overlap trials. Descriptive statistics are shown in Table 2.

**Table 2:** Descriptive statistics

Condition	Full sample	ADHD	Typically developing
*GAP*			
Latency	356.80 (91.52)	394.20 (103.96)	338.50 (79.71)
Gain	0.22 (0.08)	0.22 (0.07)	0.23 (0.09)
Variability (latency)	161.81 (94.29)	192.12 (98.48)	146.98 (89.52)
Variability (gain)	0.23 (0.09)	0.22 (0.07)	0.23 (0.10)
*OVERLAP (silent)*			
Latency	449.35 (120.63)	495.95 (111.03)	427.05 (119.77)
Gain	0.30 (0.08)	0.29 (0.09)	0.30 (0.08)
Variability (latency)	242.62 (107.77)	261.65 (104.27)	233.52 (109.35)
Variability (gain)	0.30 (0.08)	0.29 (0.09)	0.30 (0.08)
*OVERLAP (CUED)*			
Latency	426.82 (96.13)	439.31 (84.80)	421.23 (101.14)
Gain	0.30 (0.07)	0.29 (0.07)	0.30 (0.07)
Variability (latency)	201.44 (99.14)	225.77 (98.70)	190.57 (98.43)
Variability (gain)	0.30 (0.07)	0.29 (0.07)	0.30 (0.07)

## Results

### The gap effect

As expected, overlap trials resulted in longer saccadic latencies than gap trials [*F* (1,69.17) = 25.48; *p* < 0.001]. Saccadic gain was lower (closer to optimal values) in the overlap condition than in the gap condition [*F* (1,67.51) = 91.28, *p* < 0.001]. A difference between gap and overlap trials was also found on saccadic latency variability, with lower variability in the gap condition [*F* (1,68.62) = 28.77, *p* =  < 0.001, *z* = − 0.77]. In contrast, no gap effect was found for saccadic gain variability [*F* (1,131.03) = 0.17, *p* = 0.677, *z* = − 0.04]. Thus, the preliminary analyses showed a gap effect for both median saccadic latency and saccadic latency variability. Neither symptoms of ADHD, ODD nor group status (ADHD, typically developing) interacted with the gap effect for these measures (all *p* > 0.20), suggesting that the gap effect was not related to ADHD symptoms.

### Saccadic latency

To test the hypothesis that ADHD symptoms would be associated with an enhanced beneficiary effect of alerting cues, data from the silent and cued overlap conditions were analyzed in a second GLMME including a main effect of condition (silent, cued), and an interaction term between ADHD symptom level and condition.

Saccadic latencies were shorter after phasic alerting cues, but this effect was not statistically significant, [*F* (1,132.00) = 2.94, *p* = 0.089, *z* = 0.20]. However, the hypothesized interaction effect between ADHD symptoms and condition was found, [*F* (1,129.00) = 5.64, *p* = 0.019, *z* = 0.27], indicating that a greater reduction in saccadic latency after phasic alerting cues was related to higher levels of ADHD symptoms. Follow-up tests in the two conditions separately showed that ADHD symptoms were linked to longer saccadic latencies in the silent overlap condition [*F* (1,63.00) = 5.46, *p* = 0.048, *z* = 0.41], but not in the cued condition [*F* (1,63.00) = 0.57, *p* ≥ 0.50, *z* = − 0.12]. A secondary analysis with group (ADHD, typically developing) resulted in highly similar results. First, an interaction effect between group and condition was found [*F* (1,68.76) = 4.48, *p* = 0.038, *z* = 0.48], driven by a larger relative reduction in saccadic latencies on cued than on silent trials in the ADHD group. Follow-up analyses showed that saccadic latencies were longer in children with ADHD than in the typically developing group on silent [*F* (1,67.00) = 10.60, *p* = 0.004, *z* = 0.74], but not on cued trials [*F* (1,68.00) = 0.54, *p* ≥ 0.59, *z* = 0.17]. Group wise analyses showed a trend towards decreased saccadic latency in the ADHD group after phasic alerting cues [*F* (1,22.86) = 4.46, *p* = 0.092, *z* = − 0.48], but not in typically developing children [*F* (1,45.69) ≤ 0.01, *p* ≥ 0.50, *z* ≤ 0.01]. To sum up, both the dimensional and the categorical analyses showed an association between the ADHD-symptom dimensions and a decrease in saccadic latency after phasic alerting cues. No main or interaction effects involving ODD and the phasic alerting effect were found (*Supplementary materials)*.

### Saccadic gain

No significant main effect of alerting cue was found on saccadic gain [*F* (1,53.91) = 1.49, *p* = 0.228, *z* = 0.13] and similarly there was no significant interaction effect between ADHD symptom level and saccadic gain [*F* (1,66.22) = 0.08, *p* = 0.784]. Further, there was no main effect of group [*F* (1, 67.28) = 0.52, *p* = 0.472, *b* = − 0.16], and no interaction effect between group and condition [*F* (1,66.07) = 0.00, *p* = 0.951, *z* = − 0.02]. No main or interaction effects involving ODD symptoms and the phasic alerting effect were found (*Supplementary materials)*.

### Pupil dilation

As can be seen in Fig. 2, alerting cues elicited larger pupil dilation than the silent conditions (all *p* < 0.001). No significant relation was found between the pupil dilation response to phasic alerting cues and symptoms of ADHD [*F* (1,68.00) = 0.31, *p* = 0.582, *z* = 0.08] or ODD [*F* (1, 68) ≤ 0.01; *p* = 0.99].

### Variability of saccadic latency and gain

Across conditions, ADHD symptoms were associated with higher variability of saccadic latency [*F* (1,71.00) = 6.45, *p* = 0.013, *z* = 0.44], but not with variability of saccadic gain [*F* (1,70.00) = 1.80, *p* = 0.184, *z* = 0.02]. Similarly, variability of saccadic latency was higher in children with ADHD than in children with typical development [*F* (1,71.00) = 8.53, *p* = 0.005, *z* = 0.68], whereas no group difference was found on gain variability [*F* (1,70.00) = 1.46, *p* = 0.231, *z* = 0.24]. Within the overlap condition, cued trials resulted in higher variability than silent trials [*F* (1,66.35) = 7.12, *p* = 0.010, *z* = 0.39]. There were no significant interactions between ADHD symptoms and condition for either latency [*F* (1,69.50) = 0.64, *p* = 0.426, *z* = 0.12] or gain variability [*F* (1,68.14) = 0.03, *p* = 0.860, *b* = 0.02]. Similarly, group status did not interact with condition for either latency [*F* (1,66.03) ≤ 0.01, *p* = 0.950, *z* = − 0.02], or gain variability [*F* (1,66.60) = 0.34, *p* = 0.562, *z* = 0.13]. No main or interaction effects of ODD symptoms and variability of saccadic latency or gain were found (*Supplementary materials*).

## Discussion

Influential theories suggest that disrupted arousal processes underlie ADHD symptoms [18, 21, 24]. The aim of the present study was to deepen our understanding of the relation between arousal and ADHD symptoms by examining the effects of short-term increases in arousal induced by auditory warning signals (phasic alerting cues) on saccadic reactions in children with and without ADHD.

As noted in the introduction, disrupted arousal processes in other clinical conditions have been associated with enhanced sensitivity to phasic alerting effects and warning signals more generally. Therefore, we hypothesized that ADHD symptoms would be associated with greater reduction of saccadic latency following phasic alerting cues. Both dimensional analyses and group comparisons supported this prediction.

As expected, higher levels of ADHD symptoms were associated with slower saccadic reaction times in the absence of phasic alerting cues. Indeed, this effect has been reported in a number of previous studies (e.g. [11, 14, 55, 56].). Importantly, however, our results add novelty to these findings by showing that phasic alerting cues normalized this visual attention pattern by decreasing saccadic latencies in the ADHD group, but this effect was not observed in typically developing children. This interesting effect supports our hypothesis that, similar to other clinical populations with known tonic hypo-arousal, children with high levels of ADHD symptoms should benefit more from alerting effects compared to nonclinical populations. We also hypothesized that alerting cues would result in increased pupil dilation, an index of activity in the LC-NE system. This hypothesis was also supported, suggesting that children with ADHD benefitted from alerting cues because of an increase in LC-NE mediated arousal.

The hypothesis that phasic alerting cues would further lead to a more optimal saccadic gain in children with ADHD was not supported. Although, we were able to replicate the finding that ADHD symptoms are associated with increased variability in saccadic latency, variability was not affected by phasic alerting.

Our findings are novel in two important ways. First, they demonstrate that children with ADHD are hyper responsive to phasic alerting cues, and secondly, they suggest that this effect is related to an increase in pupil dilation. As described in the introduction, several theories of ADHD suggest that the disorder symptoms are resulting from tonic hypo-arousal [18, 27, 57, 58]. Our findings support this theory by showing that, similar to other populations with attenuated arousal, children with ADHD are hyper responsive to phasic alerting cues [59].

Previous studies have suggested that cognitive performance is improved by exposure to white noise in children with ADHD [36, 37]. These studies have examined performance during longer periods of white noise. Our results demonstrate that short-term increases in arousal can affect attention and behavior in children with ADHD on a very short time scale, and suggest that pupil dilation is a promising marker for studying these effects. Thereby, our results may well have high clinical value in that the observed positive effects could be used in the development of new treatment and intervention programs. Further, a recent study using a similar paradigm reported that children with autism spectrum disorder did not differ from controls in the phasic alerting effect, but were slower to shift their gaze in the overlap condition [43]. This suggests that studies of the phasic alerting effect and the gap/overlap task may be valuable in discriminating between ADHD and ASD. The dimensional analyses in the present study suggest that the effect of phasic alerting cues on saccadic latencies is independent of coexisting ODD symptoms. This is potentially important, since ADHD and ODD symptoms are highly comorbid, and differential diagnosis is often challenging [1]. Our results are consistent with other studies suggesting that manual reaction time variability can be reduced in individuals with ADHD by external incentives as well as increased stimulus presentation speed, manipulations which have been hypothesized to increase arousal [22, 35].

Some limitations of this study should be mentioned. First, the clinical sample is relatively small, meaning that the results should be replicated in larger samples. Secondly, the diagnosis of ADHD was not confirmed in an independent clinical examination. However, the fact that both our dimensional and categorical analyses yielded highly consistent results suggests that this would not be a serious confounder. Children with ADHD and typically developing controls did not differ in IQ in the present study. This means that the results are not likely to be explained by group differences in general cognitive functioning. However, since ADHD is associated with lower IQ at the population level [60], further studies are needed to determine whether the results of the present study generalizes to cognitively more low-functioning ADHD-samples. An interesting question for future research is to examine phasic alerting effects on more fine-grained oculomotor metrics believed to be affected in ADHD, such as the rate of microsaccades, which can only be studied using eye trackers with higher temporal resolution [61].

Futures studies should also examine whether phasic alerting effects are linked to treatment outcome for ADHD. As noted in the introduction, the therapeutic effect of stimulant and nonstimulant pharmacological treatments for ADHD has been linked to changes in noradrenergic activity. Future studies should therefore examine alerting effects in children with ADHD both with and without medication.

## Conclusion

The current study is the first to show that slow saccadic latencies, long known to be associated with ADHD, can be normalized by arousal-inducing auditory alerting effects. These findings contribute novelty to our understanding of the interaction process between arousal and attentional mechanisms in ADHD. As such, we believe that our study carries clear empirical and clinical value of significance for the field of developmental psychopathology.

## Electronic supplementary material

Below is the link to the electronic supplementary material.

Supplementary file1 (DOCX 19 kb)
