# Supplementary material for: Can auditory warning signals normalize eye movements in children with ADHD?
Source: Eur Child Adolesc Psychiatry. 2020 Feb 1;29(12):1635–44. doi: 10.1007/s00787-020-01484-w (PMC7641930; doi:10.1007/s00787-020-01484-w)
Supplement: Supplementary file 1 — Supplementary file1 (DOCX 19 kb) [file 787_2020_1484_MOESM1_ESM.docx]

|  |  |
| --- | --- |

Supplementary Materials

*Table S1* shows results from GLMMEs of the relation between saccadic latencies and ADHD symptoms reported in the *Results* section without ODD symptoms as covariate. For details, see *Statistical Analysis*.

*Table S1*. Relation between saccadic latency and ADHD symptoms without ODD as covariate

|  | ***z*** | ***95% CI*** | ***F (df)*** | ***P*** |
| --- | --- | --- | --- | --- |
| GAP EFFECT |  | | | |
| Gap Type (Gap, Overlap) | 0.78 | 0.58 0.96 | F (1,114.22) = 78.94 | **<.001***** |
| ADHD (main effect) | 0.17 | 0.00 0.33 | F (1,67.57) = 4.07 | **.048*** |
| Gap Type x ADHD | 0.004 | -0.01 0.02 | F (1,114.62) = 0.05 | .823 |
| ALERTING EFFECT |  | | | |
| Condition (Cued, Silent) | 0.20 | -0.03 0.43 | F (1,69.34) = 4.78 | **.032*** |
| ADHD (main effect) | 0.12 | -0.07 0.31 | F (1,70.92) = 1.51 | .223 |
| Condition x ADHD | 0.03 | 0.00 0.05 | F (1,69.59) = 5.62 | **.021*** |
| CUED TRIALS |  | | | |
| ADHD | 0.03 | -0.17 0.24 | F (1,68.00) = 0.11 | .739 |
| SILENT TRIALS |  | | | |
| ADHD | 0.30 | 0.06 0.53 | F (1,68.00) = 6.14 | **.032*ǂ** |

* p <.05; *** p <.001;

**ǂ** Bonferroni corrected

*Table S2 -S3* show results for ODD symptoms in the GLMMEs reported in the main analysis (*see Statistical Analysis* and *Results*) for details.

*Table S2*. Relation between saccadic latency and ODD symptoms

|  | ***z*** | ***95% CI*** | ***F (df)*** | ***P*** |
| --- | --- | --- | --- | --- |
| GAP EFFECT |  | | | |
| ODD (main effect) | 0.17 | 0.00 0.33 | F (1,67.57) = <.01 | .995 |
| Gap Type x ODD | -0.04 | -0.23 0.14 | F (1, 69.77) = 0.05 | .823 |
| ALERTING EFFECT |  | | | |
| ODD (main effect) | 0.12 | -0.07 0.31 | F (1,70.92) = 1.51 | .223 |
| Condition x ODD | 0.01 | -0.02 0.02 | F (1,69.64) = 0.28 | .598 |
| CUED TRIALS |  | | | |
| ODD | 0.21 | -0.12 0.54 | F (1,68.00) = 1.60 | .211 |
| SILENT TRIALS |  | | | |
| ODD | -0.16 | -0.52 0.20 | F (1,68.00) = 0.77 | .383 |

*Table S3*. Relation between saccadic gain and ODD symptoms

|  | ***z*** | ***95% CI*** | ***F (df)*** | ***P*** |
| --- | --- | --- | --- | --- |
| GAP EFFECT |  |  |  |  |
| ODD (main effect) | -0.03 | -0.28 0.22 | F (1,69.42) = 0.05 | .825 |
| ODD x gap type (gap, overlap) |  | | | |
| ALERTING EFFECT  (CUED, SILENT) | 0.04 | -0.17 0.24 | F (1, 69.77) = 0.14 | .709 |
| ODD (main effect) | -0.02 | -0.37 0.33 | F (1,68.00) = 0.01 | .914 |
| ODD x Condition (cued, silent) | 0.02 | -0.23 0.28 | F (1, 68.66) = 0.02 | .88 |
| CUED TRIALS |  | | | |
| ODD (main effect) | -0.07 | -0.38 0.24 | F (1,68.00) = 0.18 | .669 |
| SILENT TRIALS |  |  |  |  |
| ODD (main effect) | -0.02 | -0.37 0.33 | F (1,68.00) = 0.01 | .914 |

*Table S4*. Relation between variability of saccadic latency and gain and ODD symptoms

|  | ***z*** | ***95% CI*** | ***F (df)*** | ***P*** |
| --- | --- | --- | --- | --- |
| VARIABILITY OF LATENCY |  | | | |
| ODD (main effect) | -0.25 | -0.61 0.10 | F (1,71.00) = 2.05 | .156 |
| ODD x Condition (cued, silent) | -0.01 | -0.04 0.02 | F (1,68.03) = 1.46 | .231 |
| VARIABILITY OF GAIN |  |  |  |  |
| ODD (main effect) | -0.03 | -0.07 0.01 | F (1,70.00) = 2.14 | .148 |
| ODD x Condition (cued, silent) | 0.018 | -0.01 0.04 | F (1,70) = 1.98 | .162 |
